# High-Throughput Sequencing to Investigate Associations Between HLA Genes and Metamizole-Induced Agranulocytosis

**DOI:** 10.3389/fgene.2020.00951

**Published:** 2020-08-21

**Authors:** Anca Liliana Cismaru, Livia Grimm, Deborah Rudin, Luisa Ibañez, Evangelia Liakoni, Nicolas Bonadies, Reinhold Kreutz, Pär Hallberg, Mia Wadelius, EuDAC Collaborators, Manuel Haschke, Carlo R. Largiadèr, Ursula Amstutz

**Affiliations:** ^1^Department of Clinical Chemistry, Inselspital, Bern University Hospital, University of Bern, Bern, Switzerland; ^2^Graduate School for Cellular and Biomedical Sciences, University of Bern, Bern, Switzerland; ^3^Division of Clinical Pharmacology & Toxicology, University Hospital Basel, Basel, Switzerland; ^4^Department of Biomedicine, University of Basel, Basel, Switzerland; ^5^Clinical Pharmacology Service, Hospital Universitari Vall d’Hebron, Department of Pharmacology, Therapeutics and Toxicology, Fundació Institut Català de Farmacologia, Autonomous University of Barcelona, Barcelona, Spain; ^6^Clinical Pharmacology and Toxicology, Department of General Internal Medicine, Inselspital, Bern University Hospital, University of Bern, Bern, Switzerland; ^7^Institute of Pharmacology, University of Bern, Bern, Switzerland; ^8^Department of Hematology and Central Hematology Laboratory, Inselspital, Bern University Hospital, University of Bern, Bern, Switzerland; ^9^Charité - Universitätsmedizin Berlin, Corporate Member of Freie Universität Berlin, Humboldt-Universität zu Berlin, and Berlin Institute of Health, Institut für Klinische Pharmakologie und Toxikologie, Berlin, Germany; ^10^Department of Medical Sciences, Clinical Pharmacology and Science for Life Laboratory, Uppsala University, Uppsala, Sweden

**Keywords:** drug-induced agranulocytosis, metamizole (dipyrone), HLA, pharmacogenetics, high-throughput sequencing, next generation sequencing, genetic association studies

## Abstract

**Background and Objective:** Agranulocytosis is a rare and potentially life-threatening complication of metamizole (dipyrone) intake that is characterized by a loss of circulating neutrophil granulocytes. While the mechanism underlying this adverse drug reaction is not well understood, involvement of the immune system has been suggested. In addition, associations between genetic variants in the Human Leukocyte Antigen (HLA) region and agranulocytosis induced by other drugs have been reported. The aim of the present study was to assess whether genetic variants in classical HLA genes are associated with the susceptibility to metamizole-induced agranulocytosis (MIA) in a European population by targeted resequencing of eight HLA genes.

**Design:** A case-control cohort of Swiss patients with a history of neutropenia or agranulocytosis associated with metamizole exposure (*n* = 53), metamizole-tolerant (*n* = 39) and unexposed controls (*n* = 161) was recruited for this study. A high-throughput resequencing (HTS) and high-resolution typing method was used to sequence and analyze eight HLA loci in a discovery subset of this cohort (*n* = 31 cases, *n* = 38 controls). Identified candidate alleles were investigated in the full Swiss cohort as well as in two independent cohorts from Germany and Spain using HLA imputation from genome-wide SNP array data. In addition, variant calling based on HTS data was performed in the discovery subset for the class I genes *HLA-A*, -*B*, and -*C* using the HLA-specific mapper *hla-mapper*.

**Results:** Eight candidate alleles (*p* < 0.05) were identified in the discovery subset, of which *HLA*-*C^∗^04:01* was associated with MIA in the full Swiss cohort (*p* < 0.01) restricted to agranulocytosis (ANC < 0.5 × 10^9^/L) cases. However, no candidate allele showed a consistent association in the Swiss, German and Spanish cohorts. Analysis of individual sequence variants in class I genes produced consistent results with HLA typing but did not reveal additional small nucleotide variants associated with MIA.

**Conclusion:** Our results do not support an HLA-restricted T cell-mediated immune mechanism for MIA. However, we established an efficient high-resolution (three-field) eight-locus HTS HLA resequencing method to interrogate the HLA region and demonstrated the feasibility of its application to pharmacogenetic studies.

## Introduction

Metamizole (dipyrone) is a non-opioid pro-drug with analgesic and antipyretic properties that has been used in clinical practice since the early 1930s (Hinz et al., 2016). Despite an overall good efficacy and low gastrointestinal toxicity compared to other medications (Arellano and Sacristán, 1990; Andrade et al., 1998; Sánchez et al., 2002; Blaser et al., 2015), exposure to this drug has been associated with the risk of agranulocytosis, a rare and potentially fatal blood dyscrasia characterized by a decrease in circulating neutrophil granulocytes (Blaser et al., 2017). Previous studies have reported widely varying risk estimates for metamizole-induced agranulocytosis (MIA) such as 1:1500 prescriptions in Sweden and 1:1’000’000 inhabitants and year in Spain (Hedenmalm and Spigset, 2002; Ibáñez et al., 2005). Currently, national regulations worldwide differ concerning metamizole use (Van der Klauw et al., 1999; Ibáñez et al., 2005; Basak et al., 2010) with market withdrawal in countries such as the United States, the United Kingdom, Denmark, and Sweden, whereas it remains a frequently used prescription drug in other countries like Germany and Switzerland, or even readily available over-the-counter in countries like China, Mexico, and Brazil (Chan and Chan, 1996; Hedenmalm and Spigset, 2002; Blaser et al., 2015; Stammschulte et al., 2015; Miljkovic et al., 2018).

The underlying mechanisms by which metamizole induces agranulocytosis have not yet been fully elucidated. Similarly, risk factors that may render some patients more susceptible to this adverse drug reaction are poorly understood and there are no effective strategies to prevent the occurrence of MIA. Previous studies have suggested that the mechanism of toxicity may involve an immunological component, including T-cell mediated elimination of neutrophils (Claas, 1996; Uetrecht, 1996; García-Martínez et al., 2003; Andres et al., 2009). Given their role in cell-mediated innate and adaptive immunity, an association of specific human leukocyte antigen (HLA alleles), as indicated by a small previous study (Vlahov et al., 1996), may provide evidence of such an immune-mediated mechanism. Indeed, agranulocytosis induced by several other drugs such as thiamazole (methimazole), clozapine and sulfasalazine has recently been found to be associated with specific HLA alleles such as *HLA-B^∗^27:05, HLA-B^∗^08:01*, *HLA-A^∗^31:01* and *HLA-DQB1* alleles, respectively (Hallberg et al., 2016; Legge et al., 2017; Wadelius et al., 2018). Furthermore, the frequencies of HLA alleles can vary between populations and consequently so could the susceptibility to associated adverse drug reactions (Lucena et al., 2011). A recent commentary suggested the possibility of an increased susceptibility to MIA in the British, Irish and Scandinavians compared to other populations, which could be mediated by specific HLA alleles with a higher frequency in these populations (Shah, 2018). So far, larger studies investigating possible associations between genetic variation in the HLA region and MIA are lacking and are sorely needed to improve our knowledge of the mechanism underlying the hematological complications of pharmacotherapy with metamizole and to attempt to minimize the risk of MIA in the near future.

The human leukocyte antigen (HLA) region stands out from the rest of the human genome by virtue of its exceptional level of polymorphism and high gene density (Shiina et al., 2009; Robinson et al., 2015). A wealth of associations between the HLA region and adverse drug reactions (ADRs) have been reported, yet the identification of underlying causal variants has been handicapped by the strong linkage disequilibrium (LD) in this region (Shiina et al., 2009; McCormack et al., 2011; Alfirevic et al., 2012; Hosomichi et al., 2013). With the advent of high-throughput sequencing (HTS), it is now possible to profile HLA alleles at unprecedented resolution and reduced ambiguity and analyze full-length genes including intronic regions, potentially enabling deeper insights into the etiopathogenesis of HLA-ADR associations (Kishore and Petrek, 2018).

While several commercial HTS-based HLA typing kits are available, their cost may be prohibitive for population-based studies in a research context. Alternatively, non-commercial primers to amplify full-length HLA genes have been described (Shiina et al., 2012; Hosomichi et al., 2013; Ehrenberg et al., 2017), as well as several freely available bioinformatics software tools to achieve HLA typing from HTS reads (Warren et al., 2012; Bai et al., 2014; Nariai et al., 2015; Kawaguchi et al., 2017; Lee and Kingsford, 2018). Previously published non-commercial HLA HTS methods (Hosomichi et al., 2013; Ehrenberg et al., 2017) used the tagmentation approach during sequencing library preparation, which may lead to a biased pattern of coverage, such as an underrepresentation of GC-rich segments in introns 2 and 3 of *HLA-A* (Lima et al., 2019). Potentially, this bias may be reduced with alternative fragmentation approaches.

Overall, while the literature is highly enthusiastic about HTS-based HLA typing (Carapito et al., 2016; Kishore and Petrek, 2018), less attention has been brought to novel challenges that go hand in hand with these new methods. The vast degree of polymorphism of HLA genes has spawned new bioinformatics challenges associated with profiling HLA HTS data, such as identifying the best match of sequence reads to highly similar reference sequences (e.g., different alleles of the same gene as well as conserved regions between genes) and the lack of reference sequences encompassing the full genomic sequence for many alleles, which have recently been summarized (Klasberg et al., 2019). In this study, we assessed the feasibility of high-throughput sequencing of eight full-length classical HLA genes using non-commercial primers and evaluated freely available bioinformatics tools to study classical HLA genes at the level of individual alleles as well as individual small nucleotide variants (SNVs) to investigate potential associations between variation in these genes and metamizole-induced agranulocytosis.

## Materials and Methods

### Participants and Setting

Patients for this retrospective observational case-control study were recruited at two Swiss University Hospital (Basel and the Inselspital Bern University Hospital). Patients diagnosed with new-onset neutropenia or agranulocytosis while under metamizole therapy between 2005 and 2017, metamizole-tolerant control patients, as well as healthy population controls were included. Collection of biological materials and clinical information was undertaken with a written informed consent and following the principles of good clinical practice according to the Declaration of Helsinki. This study was approved by the local ethics committees (“Ethikkommission Nordwest- und Zentralschweiz” and “Kantonale Ethikkommission Bern,” BASEC protocol number 2015–00231). Clinical data was collected as described previously (Rudin et al., 2019c).

Cases were restricted to ≥18 years of age and development of neutropenia (absolute neutrophil count < 1.5 × 10^9^/L) within at least 1 day after the first intake or at the latest 2 weeks after discontinuing metamizole. Neutrophil counts prior to metamizole intake were not available for the majority of cases. Metamizole-tolerant control patients had received metamizole treatment with a daily dose of at least 500 mg for at least 28 consecutive days without any reported drug-related hematological complications. Population controls with no prior exposure to metamizole were recruited via advertisement in regional print or online media.

Out of the total of 45 cases having met the abovementioned inclusion criteria, a discovery subset of 31 metamizole-induced agranulocytosis/neutropenia (MIA/MIN) patients was selected for HTS analysis based on clinical factors supporting metamizole as the most likely causing agent. Selection was not based on the absolute neutrophil count (ANC) but on the lowest number of potentially confounding factors such as comorbidities (i.e., ongoing infectious diseases such as HIV), chemotherapy, and immunosuppressive therapy with cytotoxic drugs that may have contributed to agranulocytosis or neutropenia (Hallberg et al., 2016). Due to limits of sequencing library multiplexing, two of the originally selected cases could not be included for HTS analysis. In addition, the HTS discovery subset included all metamizole-tolerant individuals of European ancestry (*n* = 38). This discovery subset was used to obtain high-resolution HLA typing for a cost-effective discovery of potential candidate alleles associated with MIA/MIN.

For follow-up of candidate alleles, HLA imputation data from the full Swiss cohort, including the 14 cases not selected for HTS analysis and 153 population controls was used, as well as data from 286 independent subjects recruited in Germany (12 cases and 96 controls) and Spain (31 cases and 147 controls) by investigators from the European Drug-induced Agranulocytosis Consortium (EuDAC) as described previously (Hallberg et al., 2016).

### DNA and Template Preparation

Genomic DNA from whole blood or buffy coat was extracted and purified using the QIAamp DNA Blood Maxi and Midi Kits (Qiagen) according to the manufacturer’s instructions. DNA samples from subjects of the discovery cohort were amplified at eight loci (*HLA-A*, -*B*, -*C*, -*DRB1*, -*DQA1*, -*DQB1*, -*DPA1* and -*DPB1*) by full gene-length long-range PCR (Polymerase Chain Reaction). Novel primers for *HLA-DQA1*, -*DPA1* (reverse primer), and -*DPB1* (reverse primer) were designed in-house, while a combination of previously described locus-specific primers (Shiina et al., 2012; Hosomichi et al., 2013; Ehrenberg et al., 2017) was used to amplify the other five genes (Supplementary Table S1). Each locus was amplified in 25 μL reaction volumes consisting of 50–250 ng of gDNA, 1x PrimeSTAR GXL Buffer, 0.625 U PrimeSTAR GXL Polymerase (Takara Bio Inc), 0.2 μM dNTPs and 0.08 mM of the respective forward and reverse primer mix using the PCR cycling conditions described in Supplementary Table S2. To confirm amplification, 2 μL of each PCR product were analyzed on 0.8% agarose gels. The remaining product was purified by the QIAquick PCR Purification Kit (Qiagen) and quantified with a Qubit 4 Fluorometric system (Life Technologies).

### Multiplex Library Preparation and Sequencing of the Discovery Subset

For library preparation with the QIAseq FX DNA Library Kit (Qiagen), all eight HLA amplicons were pooled in equimolar amounts (except for *HLA-DRB1*, where the total input amount was doubled due to lower coverage observed in preliminary experiments) in two amplicon pools according to their sizes (see Supplementary Table S1) resulting in two libraries per subject. Amplicon pools were enzymatically fragmented (3 min for the shorter pool “S” and 4 min for the longer pool “L”), then subjected to size-selection clean-up (GeneRead Size Selection Kit, Qiagen), uniquely dual-indexed at the subject level, purified with magnetic beads (CleanNGS Kit, GC biotech) and the resulting libraries of “S” and “L” amplicons of all subjects were pooled separately in equimolar amounts. For sequencing, one part of pooled “S” libraries (3 nM) and three parts (volume) of pooled “L” libraries (3 nM) were combined and the final library was diluted to an approximate concentration of 2 nM.

The final library pool was diluted in HT-1/5% Tris-HCL, pH8.5 with 5% PhiX spiked in and sequenced on a MiSeq instrument using the paired-end 500 cycle (2x 250bp paired-end reads) MiSeq Reagent v2 Kit (both Illumina). Libraries for two samples were sequenced as part of the protocol optimization using a MiSeq Nano v2 (2 × 250 bp paired-end reads) Kit.

The PCR amplification and library preparation protocol used in this study was adapted based on preliminary experiments using anonymized samples previously HLA typed by validated SSP-PCR based methods (LinkSeq for HLA, Linkage Biosciences; HLA-FluoGene ABC, Innotrain). Using this optimized protocol, 100% concordant results with SSP-PCR typing were obtained for *HLA-A*, -*B*, -*C*, -*DQA1*, -*DQB1*, -*DPA1*, -*DPB1* and 95% concordant results in *HLA-DRB1* in 10 investigated subjects (20 alleles total; data not shown).

### Quality Control and HLA Typing of the Discovery Subset

Quality of the obtained raw sequence reads was assessed using FastQC v.0.11.7^1^ and MultiQC v.1.5 (Ewels et al., 2016) and trimmed as described in more detail in the Supplementary Appendix using the BiomedicalWorkbench v.4.1.1 (QIAgen). Briefly, trimming and read filtering consisted of removal of Universal Illumina Adapters, removal of PCR primer sequences, and removal of reads with length < 100 bp. For each individual, paired reads from both sequencing libraries were combined for subsequent analyses.

HLA typing of the trimmed and paired sequencing reads was performed with both the freely available tool HLA-HD v.1.2.0.1 (Kawaguchi et al., 2017) using read mapping with Bowtie 2 (v.2.3.4.1) (Langmead and Salzberg, 2012) and the IPD-IMGT/HLA reference dictionary v.3.34.0 (Robinson et al., 2015), as well as with the commercial software NGSengine v.2.8.0.9796 (GenDx, IPD-IMGT/HLA database v.3.30.0). Details are provided in the Supplementary Appendix.

Typing results from HLA-HD and NGSengine were compared at three-field resolution, with the exception of *HLA-DRB1*. If typing results were discordant, allele frequency information as reported in the Allele Frequency Net Database (González-Galarza et al., 2015), as well as reported *DRB1*-*DQB1* haplotype frequencies (Maiers et al., 2007) were incorporated to infer the more likely typing result. The plausibility of *HLA-DRB1* typing results was assessed based on the presence of related genes and pseudogenes and information on known DRB haplotypes (Kotsch and Blasczyk, 2000). Additional details on how the comparisons were performed are provided in the Supplementary Methods. Genotype frequencies of the accepted three-field resolution HLA typing results were tested for deviations from Hardy-Weinberg equilibrium (HWE) using Fisher’s exact test implemented in Genepop on the web v.4.2 (Raymond and Rousset, 1995; Rousset, 2008). Depth of coverage was assessed using the mean depth of coverage in exon 2 (or exon 3 in the case of *HLA-DRB2*, which lacks exon 2) and refers to the sum of the depth of coverage of the two alleles of the respective gene and sample as reported by HLA-HD.

### Genotyping Using Genome-Wide SNP Arrays

Genome-wide genotyping was performed using the Infinium CoreExome-24 V.1.2 BeadChip (Illumina) in the 253 Swiss individuals recruited for the present study (53 cases, 39 tolerant, and 161 unexposed controls). Thirty-one agranulocytosis cases from Spain and 147 Spanish controls had previously been genotyped with the Illumina HumanOmni 2.5M chip (Illumina), while an additional 36 Spanish controls had been genotyped with the Illumina HumanOmni1-Quad 1M chip. Cases (*n* = 12) and controls (*n* = 96) from Germany had been genotyped with the Illumina HumanOmniExpress 700K. Genotype calls were generated with the GenomeStudio Software (Illumina) and coordinates were based on the Genome Reference Consortium GRCh37 (hg19) build.

Standard quality control (Turner et al., 2011; Guo et al., 2014; Alonso et al., 2015) and data management was performed with PLINK v.1.7 and v.1.9 (Purcell et al., 2007). Specifically, data was filtered individually for each cohort (Swiss, Spanish, German) on the basis of single nucleotide polymorphisms (SNP) genotype call rates (>98%), sample genotype call rates (>98%), deviation from HWE in controls (*p* < 0.001) and minor allele frequency (MAF > 1%).

We performed multi-dimensional scaling (MDS) analysis including the combined data from the Swiss and EuDAC cohorts together with data from HapMap phase III samples to identify nine and five ethnic outliers in the Swiss and the EuDAC cohorts, respectively. Based on pairwise identity-by-descent (IBD) estimation, we confirmed that three pairs of individuals in the EuDAC cohorts were closely related. One individual from each pair was randomly selected to be excluded from subsequent analyses. MDS was also applied to variants in the HLA region to investigate potential global genetic differences between the cohorts in this region (see more detailed description in the Supplementary Appendix).

### Imputation of SNPs and HLA Types From Genome-Wide SNP Array Data

SNP imputation was performed by minimac3 on the Michigan Imputation Server (Das et al., 2016) using the Haplotype Reference Consortium (HRC 1.1 2016) reference panel (McCarthy et al., 2015) on the basis of pre-imputation SNP-level validated data^2^ from each cohort (see Supplementary Table S3).

HLA types of the five genes with at least one identified candidate allele (*HLA*-*B*, -*C*, -*DQA1*, -*DQB1* and -*DRB1*) were imputed using HIBAG (Zheng et al., 2014) with the published parameter estimates from the European reference population based on hg19 (Zheng et al., 2014) in the three independent cohorts. HLA imputation of the Swiss cohort was performed using imputed SNPs with *R*^2^ ≥ 0.9 to increase the number of SNPs used in the imputation model. Unimputed SNPs were used to impute HLA types of the German and Spanish cohorts, since a higher proportion of SNPs of the imputation model were directly genotyped. HLA imputation accuracy was estimated using the samples from the discovery subset that had HLA typing results available from both HTS and HLA imputation assuming results from HTS as the correct typing.

### Investigation of Individual Variants in Classical HLA Class I Genes From HTS Data in the Discovery Subset

Obtaining reliable variant calls in HLA genes has proved to be challenging with mapping bias impeding the accuracy of genotype calls from short reads (Brandt et al., 2015). To address this challenge, *hla-mapper* (Castelli et al., 2018) [v.2.3, database v002.1, default parameters plus the flags –noclean and –multiple_hits_MQ0; bwa v.0.7.17 (Li and Durbin, 2010) and SAMtools v.1.8 (Li et al., 2009)] was used to map the trimmed reads to the respective HLA class I genes of the human reference genome build hg19. Per-sample variants from the respective BAM files of *HLA-A*, -*B*, and -*C* were called using Genome Analysis Toolkit (GATK) v.4.1.3.0 (Poplin et al., 2017) in GVCF mode with default parameters considering chromosome 6 of hg19 as reference. Per-sample GVCFs were consolidated using GATK GenomicsDBImport and jointly genotyped using GATK GenotypeGVCFs producing a set of jointly called SNPs and Indels. Variants were annotated using bcftools (SAMtools) with the pattern ‘CHR:POS:REF:ALT.’

Variants were converted to PLINK BED files using PLINK v.1.9 retaining only biallelic sites. Biallelic variants were filtered on the basis of variant call rate (removing variants missing in > 1 sample), minor allele frequency (removing variants with < 4 minor variant allele count), and deviations from HWE in tolerant controls (removing variants with HWE exact test *p* < 0.001).

### Statistical Analysis

Statistical analyses of the HTS typing results in the discovery subset were performed using the R language for statistical computing v.3.5.3 (R Development Core Team,, 2019). Individual alleles at both three- and two-field resolution were tested for association with metamizole-induced neutropenia/agranulocytosis in a two-sided allelic Fisher’s exact test. Only alleles with at least four observations were included in the association analyses. Given the exploratory nature of this discovery analysis, unadjusted *p*-values were reported and alleles with *p* < 0.05 were selected as candidate alleles.

Candidate alleles were subsequently tested in the full Swiss and in the Spanish and German EuDAC cohorts to assess replication of these candidate associations. Statistical analyses were performed as described above but only at two-field resolution available from HLA imputation using a two-sided allelic Fisher’s exact test and using the R language for statistical computing v.3.6.1 (R Development Core Team,, 2019). In the Swiss cohort, candidate alleles were investigated in the full cohort, as well as with agranulocytosis cases only (ANC < 0.5 × 10^9^/L; *n* = 30). Association results were visualized using the R package forestplot (Gordon and Lumley, 2019). For replication analyses, the significance threshold was adjusted to the number of investigated candidate genes, i.e., *p* < 0.01 was considered statistically significant, corresponding to a Bonferroni correction for 5 independent tests.

Allelic associations of the filtered biallelic variants identified in the discovery subset as described in Section “Investigation of individual variants in classical HLA class I genes from HTS data in the discovery subset” were tested in PLINK v.1.9 using Fisher’s exact test. Finally, a discovery meta-analysis of all alleles was conducted for *HLA-A, -B, -C, -DRB1, -DQA1*, and *-DQB1* including two-field and one-field association results from a two-sided allelic Fisher’s exact test for the three independent cohorts (71 agranulocytosis cases and 428 controls). The meta-analysis was performed using a sample-size-weighted fixed effect scheme with METAL (Willer et al., 2010). The analysis was based on *p*-values, taking both sample size and direction of effect relative to the effect allele into account, and testing for heterogeneity of effects between the cohorts. For this discovery meta-analysis, *p* < 1 × 10^–3^ was considered statistically significant. Given the linkage between HLA genes and particularly among class I and class II genes, this threshold was selected using the maximum number of alleles tested in any cohort for the most diverse class I and II genes, respectively, *HLA-B* (29 alleles in the Spanish cohort) and *HLA-DRB1* (20 alleles in the Spanish cohort) as an estimate for the number of independent tests. In each cohort, only alleles with at least four observations were included in the initial meta-analysis. For two alleles with the lowest meta-analysis *p*-values, for which the initial meta-analysis did not include all three cohorts due to this frequency cutoff, the meta-analysis was repeated including data from all three cohorts.

## Results

### Participants

Of the 96 cases of metamizole-induced agranulocytosis recruited, ten were excluded as follows: eight cases from the Swiss cohort (two as being ethnic outliers, two due to ongoing infectious diseases, four due to immunosuppressive therapy with cytotoxic drugs) and two cases from the Spanish cohort (suggested ethnic outliers). Out of the total 443 controls available, fifteen were excluded as follows: nine from the Swiss cohort (one was recruited twice as determined by IBD analysis, seven ethnic outliers and one with low sample call rate), two from the Spanish cohort (due to cryptic relatedness) and four from the German cohort (three ethnic outliers and one due to cryptic relatedness). As determined by the MDS analysis, the proportion of participants of European ancestry among the total number recruited for the MIA-CH cohort was thus 96.5%, of which 75.5, 74, and 74% in the case, tolerant control and unexposed control groups, respectively, reported “Swiss” as their ethnicity. Characteristics of patients and control subjects are summarized in Tables 1, 2. The discovery subset included 25 out of 30 (full Swiss cohort of MIA cases with agranulocytosis, ANC < 500/μL) and 6 out of 15 cases of neutropenia (ANC < 1500/μL), while all cases of the German and Spanish cohorts had agranulocytosis.

**TABLE 1:** Patient characteristics of the three independent cohorts.

Cohort	Swiss		EUDAC		EUDAC	
	NGS discovery		German		Spanish	
	*Cases*	*Controls*	*Cases*	*Controls*	*Cases*	*Controls*
	*N* = 31(45)	*N* = 38^‡^(191)	*N* = 12	*N* = 92	*N* = 29	*N* = 145
ANC ¡ 500/μL [%]	25[87]	NA	12	NA	29	NA
Gender, male [%]	13[42]	17[45]	4[33]	NA	6[21]	NA
Age, years [%]						
¡25	7[23]	1[3]	2[16.6]	NA	3[10.3]	NA
25-44	11[35]	6[16]	6[50]	NA	6[21]	NA
45-64	10[32]	15[39]	2[16.6]	NA	12[41.4]	NA
65-74	3[10]	9[24]	2[16.6]	NA	5[17]	NA
¿74	–	7[18]	–	NA	3[10.3]	NA
BMI, median [range]	24	28	NA	NA	NA	NA
	[19-47]	[16-39]				
Latency time*^a^/treatment duration^b^, days	17	25	33.5	NA	11.5	NA
	[1-204]	[1-5297]	[4-9855]		[1-235]	

The HTS discovery subset is shown for the Swiss cohort. ^*a*^Cases, ^*b*^controls. *Latency missing for 3 agranulocytosis cases and 1 neutropenia case. ^‡^Metamizole-tolerant controls. NA, not available or not applicable.

**TABLE 2:** Patient characteristics of the full Swiss cohort.

	Full Swiss cohort		
	*Cases*	*Tolerant Controls*	*Unexposed Controls*
	N = 45	N = 38	N = 153
ANC ¡ 500/μL	30	NA	NA
Gender, male [%]	23[51]	17[45]	72[47]
Age, years [%]			
¡25	10[22]	1[2.5]	27[17.5]
25-44	14[31]	6[16]	73[48]
45-64	12[27]	15[39.5]	53[34.5]
65-74	7[15.5]	9[24]	–
¿74	2[4.5]	7[18]	–
BMI, median [range]	24[19-47]	28[16-39]	23[18-40]
Latency time*^a^ / treatment duration^b^, days	15[1-204]	25 [5197]	NA

^*a*^Cases, ^*b*^metamizole-tolerant controls. *Latency missing for 3 agranulocytosis cases and 1 neutropenia case. NA, not applicable.

### Sequencing and HTS-Based Typing of the Discovery Subset

**FIGURE 1:**
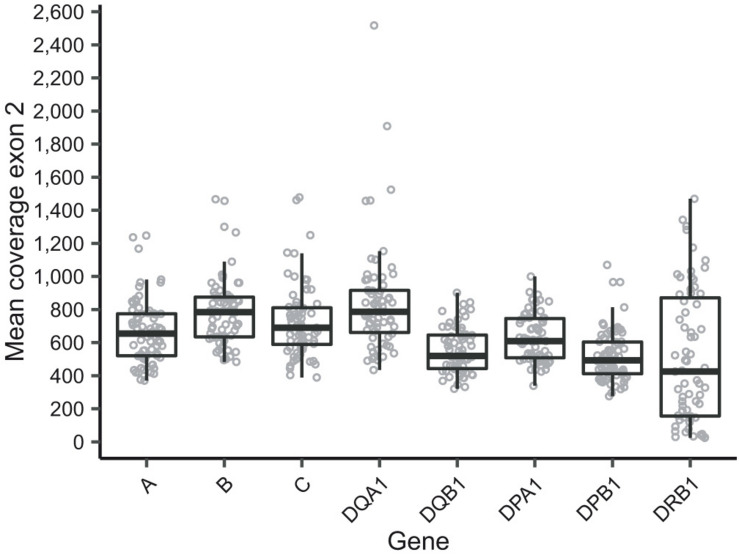
Mean depth of coverage in exon 2 as reported by HLA-HD of the eight sequenced genes of interest. Note that two cases were sequenced as part of preliminary experiments for protocol optimization and are thus not included in this figure. Additionally, samples that were sequenced but excluded for analysis are also excluded in this figure.

The current HTS method resulted in a mean depth of coverage in exon 2 of >250x in seven out of eight target genes for all samples (Figure 1). A considerably higher variation in depth of coverage between samples was observed in *HLA-DRB1*, with a mean depth as low as 24x (Figure 1). Two cases sequenced as part of preliminary method optimization with a different pooling protocol were not included in these summary statistics. Mean depth of coverage in exon 2 of *HLA-DRB1* was dependent on the allele length with lower depth observed for longer alleles (Supplementary Figure S1). In particular, alleles of the DR53 haplotype, which is characterized by the presence of *HLA-DRB4*, and to a lesser extent those of the DR52 haplotype, characterized by the presence of *HLA-DRB3*, showed lower depth of coverage (Supplementary Figure S1). In addition to the genes of interest, additional HLA loci were amplified in some samples, specifically the two *HLA-A*-related pseudogenes *HLA-H* and *HLA-Y*, the *HLA-DRB1*-related genes *HLA-DRB3*, -*DRB4*, and -*DRB5*, and pseudogenes *HLA-DRB2*, -*DRB6*, and –*DRB7* (Supplementary Figure S2). The presence of *HLA-Y*, of which only *HLA-Y^∗^02:01* was typed, appeared to be linked to specific *HLA-A* alleles (Supplementary Table S4). Five out of eight genes of interest displayed balanced allele ratios > 0.7 in every sample; whereas *HLA-DQB1* and *HLA-DRB1* showed a wider range of observed allele ratios (Supplementary Figure S3).

In most instances, HLA typing results were accepted as reported by HLA-HD. Details of discordant typing results that were investigated in more detail during quality control are shown in Supplementary Table S5. In summary, 547 of 552 results were accepted from HLA-HD, of which 477 were identical at three-field resolution to the result from NGSengine (total of 483 comparisons, excluding *HLA-DRB1*), and three results were accepted from NGSengine. In addition, quality control identified allelic dropout of *HLA-DRB1^∗^07* in two samples, which was confirmed in both instances using PCR SSP-based HLA typing by the Transplantation Immunology Laboratory of the Center for Laboratory Medicine (Inselspital Bern University Hospital). No deviation from HWE was detected for any of the eight genes of interest.

### Association Analyses and Identification of Candidate Alleles in the Discovery Subset

**FIGURE 2:**
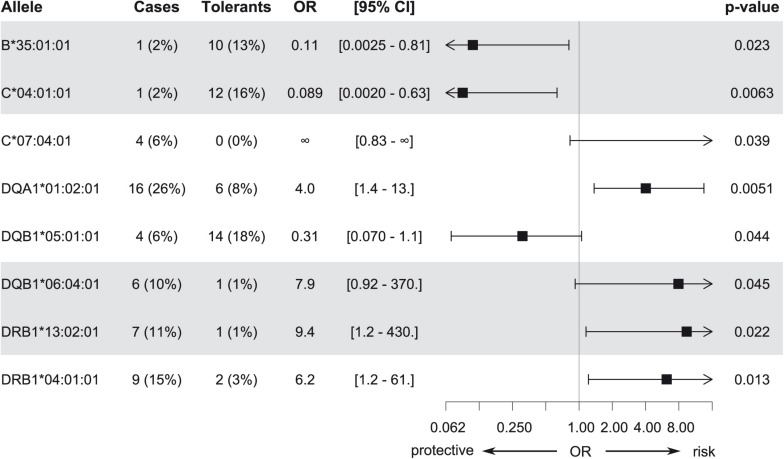
Candidate alleles with evidence for association with MIA/MIN (*p* < 0.05) in the discovery subset at three-field resolution. Odds ratios (OR), 95% confidence intervals, and unadjusted *p*-values are shown.

Association analysis of individual HLA alleles obtained with HTS typing in the discovery subset identified eight candidates for suggestive association with MIN/MIA in five different genes (Figure 2 and Supplementary Table S6). The same alleles were identified at three-field (Figure 2) and at two-field resolution (Supplementary Figure S4). In total, 73 (three-field) and 75 (two-field) alleles were tested. Allele frequencies of all alleles at three-field resolution in cases and tolerant controls of the discovery subset are summarized in Supplementary Table S6.

### Replication of Candidate Associations Using HLA Imputation

Accuracy of HLA imputation from genome-wide SNP array data in the discovery subset was >94% for four out of five imputed genes (Table 3). Moreover, the identified candidate alleles were imputed with high sensitivity and specificity in the discovery subset (Table 4).

**TABLE 3:** Accuracy of HLA imputation of the five genes where a candidate allele was identified by HTS in the discovery cohort.

Gene	Imputation accuracy
*HLA-B*	96.4% (133/138)
*HLA-C*	94.9% (131/138)
*HLA-DQA1*	97.1% (134/138)
*HLA-DQB1*	98.6% (136/138)
*HLA-DRB1*	88.4% (122/138)

*Accuracies were determined by comparing the typing results of the discovery subset, i.e., the samples for which a typing result by HTS was available, to those obtained by imputation. Number in brackets indicate the number of correctly imputed alleles and the total number of alleles imputed for each gene.*

**TABLE 4:** Sensitivity and specificity of HLA imputation for the eight candidate alleles identified by HTS in the discovery subset compared to HLA typing results of the HTS method.

Allele	Sensitivity	Specificity
B*35:01	9/11 (82%)	127/127 (100%)
C*04:01	13/13 (100%)	125/125 (100%)
C*07:04	4/4 (100%)	134/134 (100%)
DQA1*01:02	24/24 (100%)	114/114 (100%)
DQB1*05:01	18/18 (100%)	120/120 (100%)
DQB1*06:04	7/7 (100%)	131/131 (100%)
DRB1*04:01	11/11 (100%)	127/127 (100%)
DRB1*13:02	8/8 (100%)	130/130 (100%)

**FIGURE 3:**
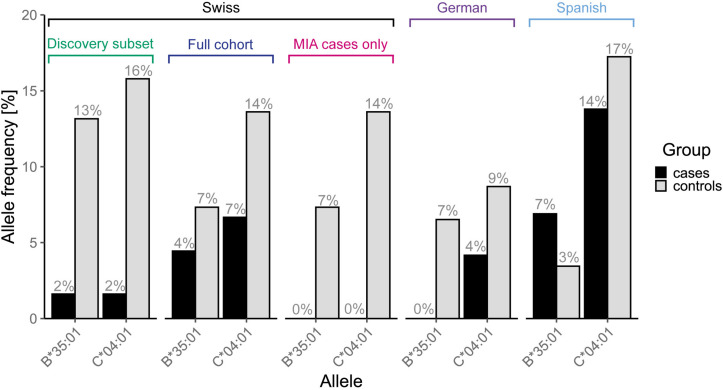
Allele frequencies of the two linked candidate alleles, *HLA-B^∗^35:01* and –*C^∗^04:01*, that showed a stronger association in the full cohort when restricting the analysis to agranulocytosis cases only, in all analyzed cohorts and subsets thereof.

**FIGURE 4:**
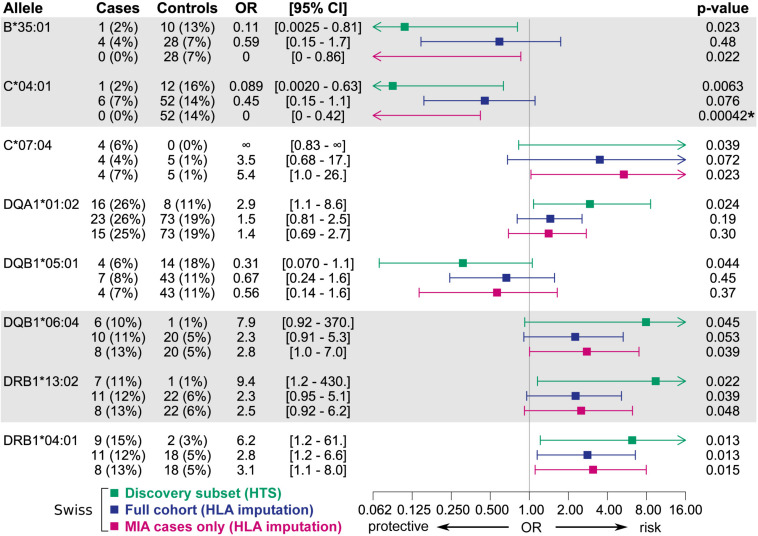
Associations of candidate alleles from the discovery subset at two-field resolution in the full Swiss cohort and in an analysis including only cases with MIA. HLA typing results obtained by HTS were used if available, else typing results obtained by HLA imputation were used. Alleles in strong linkage disequilibrium are highlighted in one contiguous gray block. *P*-values < 0.01 in the full cohort are highlighted with an asterisk.

Overall, candidate alleles showed a similar trend in the full Swiss cohort as in the discovery subset, albeit with a smaller effect size (Figure 3). However, none of the candidate alleles showed *p* < 0.01 in the full cohort, whereas a single allele (*HLA*–*C^∗^04:01)* did so when restricting the analysis to cases with MIA only. This allele, which was overrepresented in the control group, showed a stronger association with respect to MIA in the full cohort (Figure 4). *HLA*–*C^∗^04:01* was strongly linked to *HLA-B^∗^35:01* with 31 out of 32 carriers of the less common *HLA-B^∗^35:01* allele also carrying *HLA-C^∗^04:01*.

**FIGURE 5:**
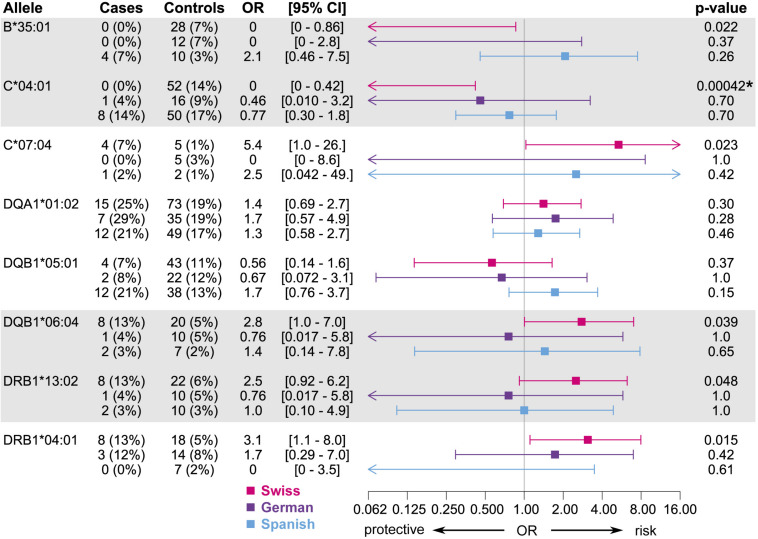
Associations of candidate alleles from the discovery subset at two-field resolution in the three independent cohorts, where cases are restricted to those meeting the criterion for agranulocytosis. HLA typing results obtained by HTS were used if available, else typing results obtained by HLA imputation were used. Alleles in strong linkage disequilibrium are highlighted in one contiguous gray block. All *p*-values < 0.01 are highlighted with an asterisk.

When expanding the analyses to the EuDAC cohorts, the general trend of the effect of these candidate alleles on MIA was not consistent in all three independent cohorts (Figure 5). The two most promising, linked candidate alleles *HLA-B^∗^35:01* and –*C^∗^04:01* showed a similarly protective trend in the Swiss and German cohorts including only cases of agranulocytosis, but not in the Spanish cohort (Figure 4). No global difference of genetic variation in the HLA region from genome-wide SNP array data was observed in Spanish versus German and Swiss controls based on MDS analysis of HLA region SNPs (Supplementary Figure 5).

### Associations of Individual Sequence Variants in Full-Length Genes

In total, 13 out of 725 tested individual variants obtained by HTS in the three full-length classical HLA class I genes were identified as candidates for an association with MIA/MIN (*p* < 0.05) (Table 5). Over 50% of carriers of the alternate allele for 12 out of 13 candidate variants also carried the candidate allele *HLA-C^∗^04:01* and 100% of carriers of eight of these variants also carried *HLA-C^∗^04:01*. The remaining candidate variant was carried together with the candidate allele *HLA-C^∗^07:04* in all carriers of the alternate allele.

**TABLE 5:** Results from Fisher’s exact test for association of individual variants in the classical class I genes *HLA-A*, -*B*, and –*C* obtained by variant calling of the HTS data.

CHR	Position	Ref	Alt	Cases	Tolerants	*P*	OR	Gene	Exon/Intron	Comment
6	31237779	C	T	1 (2%)	12 (16%)	0.0063	0.087	*HLA-C*	Exon 5	Co-occured with C*04:01:01
6	31238027	C	A	1 (2%)	12 (16%)	0.0063	0.087	*HLA-C*	Exon 4	
6	31238267	C	T	1 (2%)	12 (16%)	0.0063	0.087	*HLA-C*	Intron 3	
6	31238538	G	C	1 (2%)	12 (16%)	0.0063	0.087	*HLA-C*	Intron 3	
6	31239082	G	C	1 (2%)	12 (16%)	0.0063	0.087	*HLA-C*	Exon 3	
6	31239501	G	T	1 (2%)	12 (16%)	0.0063	0.087	*HLA-C*	Exon 2	
6	31239607	G	A	1 (2%)	12 (16%)	0.0063	0.087	*HLA-C*	Exon 2	
6	31239742	G	C	1 (2%)	12 (16%)	0.0063	0.087	*HLA-C*	Intron 1	
6	31239652	G	A	3 (5%)	15 (20%)	0.011	0.21	*HLA-C*	Intron 1	
6	31239660	G	T	3 (5%)	15 (20%)	0.011	0.21	*HLA-C*	Intron 1	
6	31237987	C	T	2 (3%)	13 (17%)	0.012	0.16	*HLA-C*	Exon 4	
6	31239057	C	T	5 (8%)	18 (24%)	0.020	0.28	*HLA-C*	Exon 3	
6	31238928	TCA	T	4 (6%)	0 (0%)	0.039	NA	*HLA-C*	Exon 3	Co-occured with C*07:04:01

*Only variants with *p* < 0.05 are shown and sorted according to *p*-value. Number of alleles and allele frequency (%) of the alternate allele (Alt) of cases and tolerant controls are indicated in the columns “Cases” and “Tolerants” respectively. Blocks of variants which are present in the same samples are highlighted in one block of the same shade of gray. If a shown variant co-occurred with a candidate HLA allele in the discovery subset, i.e., 100% of Alt allele carriers also carried the respective candidate allele, it is indicated in the column “Comment.” Genomic positions are based on hg19.*

### Discovery Meta-Analysis of Imputed HLA Alleles Across Cohorts

In the meta-analysis of all two-field and one-field resolution imputed HLA typing across all three cohorts, no alleles were significantly associated with MIA with a *p*-value of ≤1 × 10^–3^ (Supplementary Table S7). In the analysis of alleles at two-field resolution, the top allele with the smallest *p*-value was *HLA-A^∗^68:02* (*p* = 3 × 10^–3^). This result was derived only from the Spanish cohort as this allele had less than four observations in the other two cohorts. Further exploration of the data for this allele revealed that *HLA-A^∗^68:02* was not observed in any of the German or Swiss cases, and a meta-analysis including the data from these two cohorts yielded a weaker association result (*p* = 0.08, Supplementary Table S7). In the analysis of candidate alleles with one-field resolution, the allele with the smallest *p*-value was *HLA-A^∗^31* (*p* = 4.5 × 10^–3^). This result did not consider the German cohort where this allele was not observed in any of the cases and only in two controls. Also for this allele, inclusion of all three cohorts resulted in an overall weaker association (*p* = 0.01, Supplementary Table S7). When considering these results based on all data from all three cohorts, the candidate allele *HLA-C^∗^04:01* was thus the top association signal (*p* = 0.0059) also in the meta-analysis (Supplementary Table S7).

## Discussion

In this study, we investigated the association between genetic variation in HLA genes and metamizole-induced agranulocytosis/neutropenia (MIA/MIN). To our knowledge, this is the first pharmacogenetic association analysis of full-length HLA genes using an HTS approach based on non-commercial primers and utilizing freely available software tools. This exploratory analysis in a Swiss discovery subset identified eight candidate alleles in five different classical class I and II genes to be potentially associated with MIA/MIN. Candidate alleles were further investigated in an extended Swiss cohort as well as two independent European replication cohorts. However, none of these alleles were consistently associated in all three cohorts and no significant associations of other alleles were identified in a discovery meta-analysis of all cohorts, thus challenging the hypothesis of an HLA-restricted T cell-mediated mechanism behind metamizole-induced agranulocytosis.

### Metamizole-Induced Agranulocytosis and HLA Gene Variation

The possibility of certain HLA alleles conferring an increased susceptibility to MIA, which could put certain populations at a greater risk, has previously been suggested (Vlahov et al., 1996; Shah, 2018). However, in the current study, no major HLA risk allele with a strongly increased frequency among patients with MIA or MIN was detected, thus making a T cell-mediated immune mechanism restricted by a specific HLA allele unlikely. Interestingly, previous studies investigating agranulocytosis related to other drugs found strong associations with HLA risk alleles (Yunis et al., 1995; Mallal et al., 2002, 2008; Illing et al., 2012; Chen et al., 2015; Hallberg et al., 2016; Saito et al., 2016; He et al., 2017; Thao et al., 2018; Wadelius et al., 2018), some of which have been replicated in independent cohorts.

Our findings for MIA do not align with findings for other agranulocytosis-inducing drugs, raising the question whether the mechanism of inducing agranulocytosis differs between causal drugs. Indeed, our findings are consistent with recent functional studies indicating a direct toxic effect of the main metabolite of metamizole in the presence of hemin on granulocyte precursors (Rudin et al., 2019a, b). Moreover, a recent *in vitro* investigation of patient-derived peripheral blood cells did not detect any activation of the adaptive immune system by either metamizole or its main metabolite (Dina Manuel, 2019). To our knowledge, only one earlier study investigated HLA variation in the context of metamizole-induced agranulocytosis (Vlahov et al., 1996), reporting some associated alleles. However, this study included only five cases of MIA, and the reported alleles did not show the same effect in the cohorts analyzed here, which may be explained by the very limited sample size of the previous investigation. Taken together, these results thus do not support an HLA-restricted T cell-mediated mechanism of MIA.

Of note, the candidate allele *HLA-C^∗^04:01:01* with the strongest evidence for association showed a putative protective effect, i.e., it was observed at very low frequency or was absent in MIA cases. Interestingly, a similar trend was observed for this candidate allele and the linked allele *HLA-B^∗^35:01* in the German, but only to a much weaker extent in the Spanish cohort. While no global difference was observed between Spanish, German and Swiss individuals by MDS analysis of SNPs in the HLA region, we cannot exclude that individual alleles may differ in their frequency between these populations, resulting in differences in the observed associations with MIA as reported for other idiosyncratic ADRs (Lucena et al., 2011). Nevertheless, given the exploratory nature of our analyses in the discovery subset, the identified candidates are expected to include false positive results. Further validation in additional independent study populations would thus be required to assess whether *HLA-C^∗^04:01*, which also yielded the top association signal in the meta-analysis of all alleles in all cohorts, or any of the other putative candidates confer true associations. However, even if such a protective effect of *HLA-C^∗^04:01* were indeed present, its underlying mechanisms would differ from a T cell mediated mechanism mediated by a specific HLA allele. Importantly, an association detected in classical HLA genes may also result from a mechanism mediated by other genes or regulatory variants in linkage disequilibrium (LD) with HLA alleles as it is often challenging to differentiate the effect of classical HLA genes and other non-HLA genes or regulatory variants within this region (Shiina et al., 2009; McCormack et al., 2011; Alfirevic et al., 2012; Hosomichi et al., 2013).

As MIA is a rare occurrence, identifying a large number of cases for genetic association studies is challenging. Nevertheless, due to the large effect sizes reported for HLA associations with other idiosyncratic ADRs, our analyses in the Swiss discovery subset were sufficiently powered to detect associations of similar magnitude. Specifically, several such previous studies reported odds ratios (ORs) > 50 (Mallal et al., 2002; Hung et al., 2005; Daly et al., 2009) and others with somewhat weaker effect sizes still reported ORs in the range of 7–15 (McCormack et al., 2011; Wadelius et al., 2018). Given the sample size of the discovery subset, our study had sufficient power (80%) to detect associations of rarer (OR ≥ 8.5 for allele frequencies of ≥2%) and more common alleles with clinically relevant effect sizes [OR of ≥5.0 for allele frequency of ≥5% and OR of ≥3.7 for allele frequency of ≥10%; calculated using G^∗^Power v3.1 (Faul et al., 2007; Erdfelder et al., 2009)]. These minimum detectable ORs are thus lower than most previously reported associations of HLA risk alleles mentioned above.

Another challenging aspect to be noted is the ascertainment of metamizole as the causative agent for agranulocytosis and the lack of data on neutrophil counts prior to metamizole administration. Multiple medications that are commonly used concurrently with metamizole such as beta-lactam antibiotics, have also been associated with lowered neutrophil counts. However, restricting analyses to patients without such concomitant medications would result in very limited sample sizes and an associated loss in statistical power. Finally, while HLA alleles in the discovery subset were determined at higher resolution using HTS, the typing resolution in the replication cohorts based on HLA imputation was limited. While concordance of HLA imputation at two-field resolution with HTS-based typing was generally high, particularly for one of the candidate alleles (*HLA-B^∗^35:01)*, a slightly reduced sensitivity was observed. Importantly, HLA imputation accuracy is limited by the resolution of the reference data available, which is currently primarily based on traditional HLA typing methods.

### HTS-Based HLA Sequencing for Pharmacogenetic Association Studies

With the present method, we obtained three-field resolution HLA typing results, achieving higher resolution and less ambiguity compared to traditional HLA typing methods based on core exons alone or with HLA imputation. As a result, using HTS-based direct analysis of the HLA region may have benefits for future genetic association studies. In this context, our results demonstrate the feasibility of HTS-based HLA analyses in pharmacogenetic studies using non-commercial methods and data analysis tools.

The development of HTS-based HLA typing approaches has been met with great enthusiasm (Carapito et al., 2016; Kishore and Petrek, 2018) but currently faces certain limitations. Similar to other HTS-methods, our analysis included full-length genes, enabling, in principle, typing at four-field resolution. To date, however, this resolution was limited to three fields due to the limited characterization of intronic and UTR regions in the HLA reference database (Kawaguchi et al., 2017). A more complete dictionary of full-length reference sequences for HLA alleles is imperative to support full four-field resolution typing and maximize the potential of HTS-based HLA analysis.

In contrast to another recent pharmacogenetic association study using HTS-based HLA typing (Nakatani et al., 2019), our analyses not only included HLA typing, but also assessed sequence variation at the level of SNVs in full-length class I genes. Such analyses at the level of individual SNVs may complement traditional haplotype-level allele typing approaches in association studies by enabling the detection of associations for SNVs contained on multiple HLA alleles, for which analyses at the allele-level may be underpowered. Similarly, it may uncover causal variants underlying known haplotype-level associations with ADRs or diseases by providing effect sizes for individual sequence changes.

We restricted our present analysis of individual HLA SNVs to class I genes as *hla-mapper*, the HLA-dedicated mapping tool, was only available for these genes. Due to the extensive sequence variation in HLA genes, variant calls in the HLA region obtained from standard read mapping pipelines have been reported to show a higher error rate compared to other parts of the human genome with a reference allele bias (Brandt et al., 2015). The congruence of our variant calling results with the results of HLA typing demonstrates the feasibility of achieving sequence variation data at the level of individual SNVs with the help of publicly available HLA-specific software. However, such software is currently scarce and not available for HLA class II genes, which display a more challenging pattern of variation, including structural variants encompassing larger gene regions and pseudogenes with highly similar sequences (Klasberg et al., 2019). Thus, for a more comprehensive assessment of HLA variation at the level of SNVs, additional or updated HLA-specific mapping tools that include class II genes are needed.

The present method differs from other non-commercial HLA HTS methods (Hosomichi et al., 2013; Ehrenberg et al., 2017) in that it relies on an enzymatic fragmentation of amplicons and instead of a tagmentation approach in order to minimize coverage bias (Lima et al., 2019). This alternative library preparation approach presented here may thus be applied to long-range PCR products generated with any available primers to amplify HLA genes. Furthermore, in contrast to the method used here, some commercially available HTS-based HLA typing kits do not amplify exon 1 and parts of intron 1 of most HLA class II genes, thus leading to ambiguous three-field typing results. This omission of exon 1 likely reflects the difficulty in amplifying full-length class II genes as single amplicons with even amplification efficacy across alleles. In particular, the genomic length of *HLA-DRB1* varies considerably between alleles, primarily due to large structural variants in intron 1 (Von Salomé et al., 2007). Indeed, we observed a lower amplification efficiency of longer *HLA-DRB1* alleles leading to more pronounced allelic imbalance and some allelic dropout. Amplification efficiency of some *HLA-DRB1* alleles may be further impaired by co-amplification of *HLA-DRB4* or -*DRB2*. Here, we detected suspected allelic dropout for several samples using our quality control procedure. Nevertheless, given the low allele ratios for *HLA-DQB1* observed in a few samples, and it is possible that some additional allelic dropout was not detected. This allelic imbalance could potentially be reduced by using higher PCR primer concentrations as previously described (Hosomichi et al., 2013; Ehrenberg et al., 2017). Alternatively, for future studies, it may be advantageous to amplify these class II genes in two separate amplicons (Shiina et al., 2012).

Finally, our study highlights additional inherent difficulties in identifying primers that amplify all alleles of full-length HLA genes without amplifying closely related pseudogenes. Specifically, in addition to co-amplification of *HLA-DRB1* related genes, we also detected co-amplification of the *HLA-A* related pseudogene *HLA-Y* (Kawaguchi et al., 2017). Of note, this pseudogene is not included in any of the human reference genome sequences (primary assembly or alternate haplotype thereof), making it difficult to account for during primer design. Given that such co-amplification of pseudogene sequences may not be fully avoided for HLA genes, it is thus crucial that their presence be considered at the data analysis stage. For example, HLA-HD detected reads originating from *HLA-Y* while the version of NGSengine used in the presented analyses did not. Given these numerous challenges associated with PCR amplification of full-length HLA genes, alternative PCR-free approaches for isolating genomic target regions for subsequent HTS, such as the use of CRISPR/Cas9 (Nachmanson et al., 2018), may prove advantageous for future analysis of this genomic region.

## Conclusion

In this study, we did not find any consistent associations between HLA alleles and MIA across different cohorts by investigating variation in HLA genes in detail. In particular, no strong enrichment of distinct HLA alleles in patients with MIA was found. Our results, therefore, do not support an HLA-restricted T cell-mediated immune mechanism of MIA. Nevertheless, our study presents the feasibility of HTS-based analysis of HLA genes in the context of a pharmacogenetic association study, without the need to rely on costly commercial solutions, and highlights the need for further development of dedicated bioinformatics tools for HTS-based HLA data analysis.

## EuDac Collaborative Authors

Niclas Eriksson (Uppsala Clinical Research Center and Department of Medical Sciences, Uppsala University, Uppsala, Sweden); Mariam Molokhia (Department of Population Health Sciences, King’s College London, London, United Kingdom); Alfonso Carvajal (Centro de Estudios sobre la Seguridad de los Medicamentos, Universidad de Valladolid, Valladolid, Spain); M. Isabel Lucena (S Farmacologia Clinica, IBIMA, H Universitario Virgin de la Victoria, Universidad de Malaga, CIBERehd, Malaga, Spain); Javier Martin (Instituto de Parasitología y Biomedicina Lopez-Neyra, CSIC, Granada, Spain); Esther Sancho Ponce (Servei d’Hematologia I Banc de Sang, Hospital Genral de Catalunya, Sant Cugat del Vallès, Spain); Lourdes Vendrell (Fundació Institut Català de Farmacologia, Hospital Universitari Vall d’Hebron, Barcelona, Spain); Ramon Puig Treserra (Fundació Institut Català de Farmacologia, Barcelona, Spain); Jose Luis Caro (Banc de Sang i Teixits, Barcelona, Spain); Eduard Palou (Banc de Sang i Teixits, Barcelona, Spain); María José Herrero (Banc de Sang i Teixits, Barcelona, Spain); Esmeralda de la Banda Ledrado (Hospital Universitario Bellvitge, Barcelona, Spain); Eva Montané (Hospital Universitari Germans Trias i Pujol, Universitat Autònoma de Barcelona); Elisa Orna Montero (Institut Català d’Oncologia-Hospital Germans Trias I Pujol, Badalona, Spain); José Tomás Navarro Ferrando (Catalan Institute of Oncology; Francesc Salvador Rudilla, Banc de Sang i Teixits, Barcelona, Spain); Consuelo Pedrós Cholvi (Hospital Universitario Bellvitge, Barcelona, Spain).

## Data Availability Statement

The datasets for this article are not publicly available due to insufficient consent to share individual-level data publicly. Aggregate data (allele frequencies for HTS based typing) are included as Supplementary Material to this article for all investigated HLA genes. Requests to access individual-level datasets should be directed to the corresponding author.

## Ethics Statement

The studies involving human participants were reviewed and approved by Ethikkommission Nordwest- und Zentralschweiz and Kantonale Ethikkommission Bern, Switzerland. The patients/participants provided their written informed consent to participate in this study.

## Author Contributions

AC and LG contributed to the sample preparation, data acquisition, and analysis of the data, as well as manuscript preparation. MH and UA were key senior contributors and made substantial contributions to the study conception and design as well as interpretation of the data. DR, EL, LI, RK, and EuDAC collaborators recruited the study participants and collected the clinical data and biological material. CL provided input on the statistical analysis and data interpretation. MW and PH contributed to the conception and design of the EuDAC study. All authors and EuDAC collaborators provided critical feedback to help shape the manuscript. All authors read and approved the final manuscript.

## Conflict of Interest

The authors declare that the research was conducted in the absence of any commercial or financial relationships that could be construed as a potential conflict of interest.

## Footnotes

1 https://www.bioinformatics.babraham.ac.uk/projects/fastqc/ (last accessed 04.12.2019)

2 https://www.well.ox.ac.uk/∼wrayner/tools/
