# Supplementary material for: High-Throughput Sequencing to Investigate Associations Between HLA Genes and Metamizole-Induced Agranulocytosis
Source: Front Genet. 2020 Aug 21;11:951. doi: 10.3389/fgene.2020.00951 (PMC7473498; doi:10.3389/fgene.2020.00951)
Supplement: Supplementary file 1 [file Data_Sheet_1.zip › Data Sheet 1.PDF]

Supplementary Material

1 Supplementary Methods

1.1 HTS Data Quality Control and HLA Typing in the Discovery Subset

Read trimming using Biomedical Workbench v.4.1.1 (QIAgen) consisted of three steps and was performed using default settings if not specified otherwise. Firstly, Illumina Universal Adapters were trimmed from the 3' end of the reads by specifying the reverse complement of the Universal Illumina Adapters[1] to be searched on the minus strand. Secondly, the used PCR primers were trimmed by specifying the respective primers and their reverse complement to be searched on the plus and minus strand in accordance with the manual of the Biomedical Workbench. Lastly, reads with a length < 100 bp were dropped and paired reads only were exported as fastq.gz. Reads from the two libraries of shorter and longer genes prepared with different barcodes for the same patient were merged for subsequent analyses.

Typing with HLA-HD v1.2.0.1 was performed using the IPD-IMGT/HLA reference dictionary v3.34.0 (Robinson et al., 2015) with HLA_gene.split.3.32.0.txt to also be able to type *HLA-DPA2*, *-T*, *-W*, and *-Y*, and using Bowtie 2 (v2.3.4.1) (Langmead and Salzberg, 2012) to map reads. Typing with NGSengine v2.8.0.9796 was performed with the provided IPD-IMGT/HLA database v3.30.0 with mostly default settings (Illumina) except for the region average allele threshold and the noise threshold, which were both lowered to 10% in order to be able to type alleles with a greater allele imbalance.

HLA-DRB1 was excluded from typing with NGSengine and thus also from the comparison between the two typing tools, as preliminary experiments indicated that typing results of this gene obtained with NGSengine using the described PCR primers and protocol were often discordant with reference typing results obtained from the HLA typing laboratory at the Centre for Laboratory Medicine in Bern based on a clinically validated SSP-PCR method (LinkSeq for HLA, Linkage Biosciences; HLA-FluoGene ABC, Innotrain).

If both typing results were in agreement, the three-field resolution typing result was accepted (i.e. as reported by HLA-HD). If the typing results were discordant yet both software tools reported a heterozygous typing result, the typing result with the higher reported population frequency in the Allele Frequency Net Database (González-Galarza et al., 2015) in the populations Germany Essen as well as Germany pop 2 for *HLA-DPB1* was accepted. If one reported typing result was homozygous and the other one heterozygous, which only occurred for some *HLA-DQB1* typing results, the plausibility of the typing results was assessed based on the reported *DRB1-DQB1* haplotype frequencies[2] (Maiers et al., 2007). If the heterozygous result was plausible, it was accepted. As *HLA-DRB1* alleles are associated with the presence and absence of other *HLA-DRB* (pseudo-) genes, some of which were co-amplified with the present method, plausibility of *HLA-DRB1* typing results obtained with HLA-HD

---

[1] https://support.illumina.com/bulletins/2016/12/what-sequences-do-i-use-for-adapter-trimming.html (last accessed 04.12.2019)

[2] https://bioinformatics.bethematchclinical.org/hla-resources/haplotype-frequencies/high-resolution-hla-alleles-and-haplotypes-in-the-us-population/ (last accessed 04.12.2019)

was assessed using this information (Kotsch and Blasczyk, 2000). If allelic dropout in *HLA-DRB1* was suspected due to the presence of a *HLA-DRB* pseudogene not known to be associated with the typed *DRB1* allele, the sample was typed at the HLA laboratory of the Center for Laboratory Medicine, Bern University Hospital using a clinically validated PCR-SSP-based method (FluoGene v.1.5.5.3, Innotrain).

1.2 MDS Analysis of HLA region SNPs

Analyses were performed in PLINK v.1.9. From quality-controlled, unimputed SNPs of chromosome 6 of the three independent cohorts (Swiss, German, Spanish), SNPs within the HLA region (position 29719561 – 32883508 (Gogarten et al., 2012)) were extracted. The three data sets of HLA region SNPs were merged, retaining only SNPs that were genotyped in all three cohorts. LD pruning (--indep-pairwise 50 5 0.2) was performed on the SNPs from control samples of the merged data sets, followed by MDS analysis. First four dimensions were visualized in R.

2 Supplementary Figures and Tables

2.1 Supplementary Figures

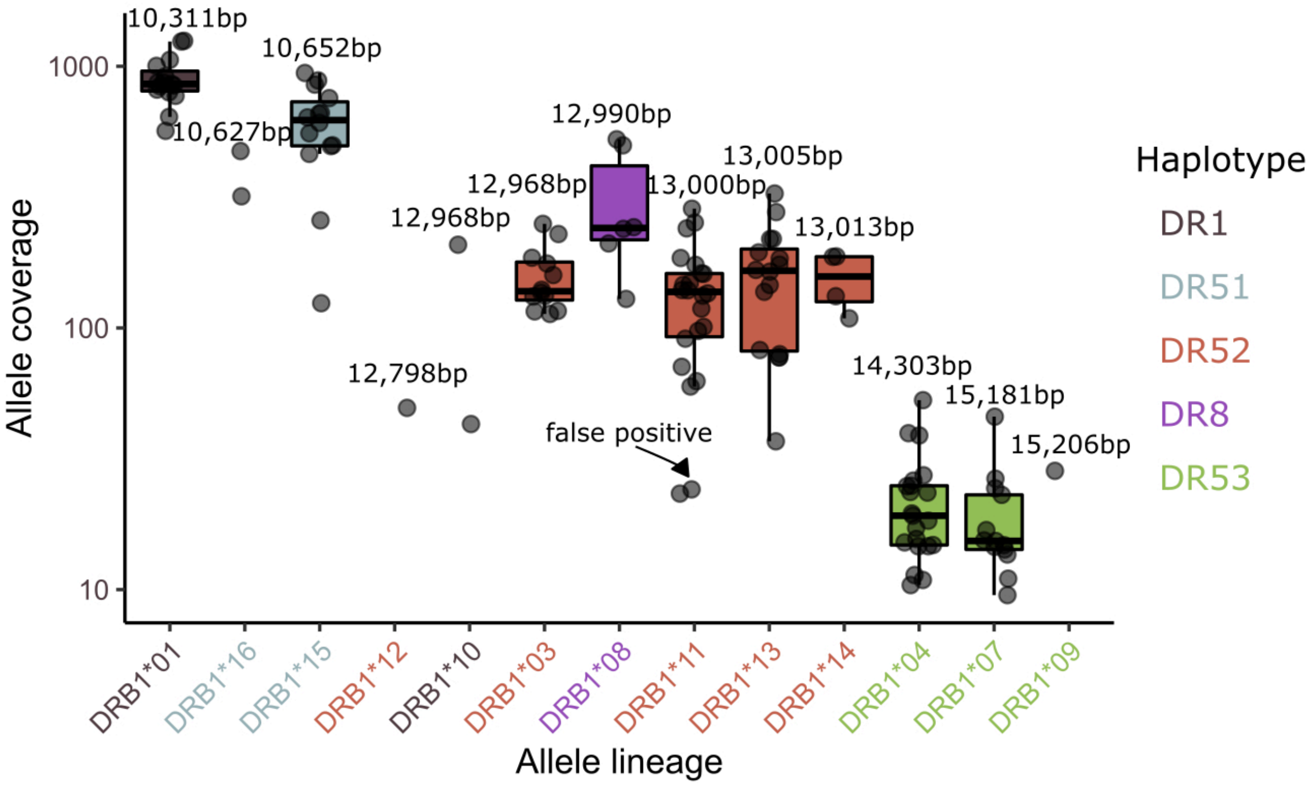

Supplementary Figure S1: Allele-specific exon 2 depth of coverage of different *HLA-DRB1* alleles grouped by allele lineage, excluding the two cases sequenced as part of preliminary experiments for method optimization. Allele lineage lengths were determined as the genomic length, from start to stop codon, of the most commonly observed allele in the discovery cohort, where a full genomic reference sequence was available in the IPD-IMGT/HLA database v3.35.0. Genomic sequences were obtained using the first available four-field allele in the database, i.e. HLA-DRB1*XX:YY:ZZ:01. Haplotype denotes the five different human DR haplotypes as described in (Kotsch and Blasczyk, 2000). Two individuals had very low coverage in their respective *HLA-DRB1*13* alleles (both around 25x). One individual (denoted false positive with an arrow) was subsequently identified not to carry an *HLA-DRB1*13* allele but a *-DRB1*07* allele, which was not typed due to allelic dropout. The second individual, on the other hand, also had a low depth of coverage for its other typed *-DRB1* allele, *-DRB1*04*. The presence of *HLA-DRB4* and *-DRB2* was concordant with obtained typing results for this subject.

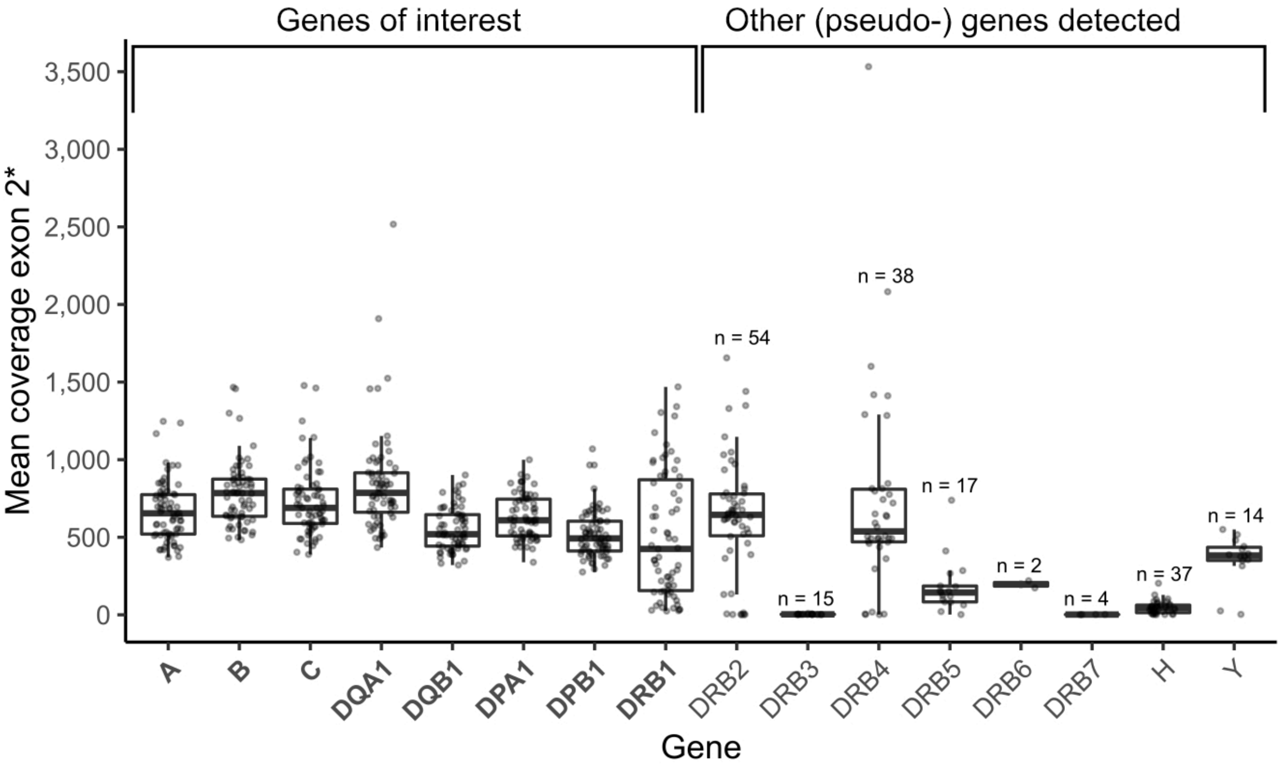

Supplementary Figure S2: Overview of the mean depth of coverage in exon 2 (or exon 3 for *HLA-DRB2*, which lacks exon 2) of HLA genes as reported by HLA-HD, i.e. excluding two cases sequenced as part of preliminary experiments for method optimization. Typing results with incomplete coverage in exon 2 or exon 3, respectively, as reported by HLA-HD, only concerned non-target genes and were excluded. The eight genes of interest (highlighted in bold) are depicted on the left while additional loci, which were typed with HLA-HD, are shown on the right. Outliers with low coverage in *HLA-DRB2*, -*DRB4*, and -*Y*, which were regarded as negative results, are included. Only data from samples included in the reported association analyses are shown.

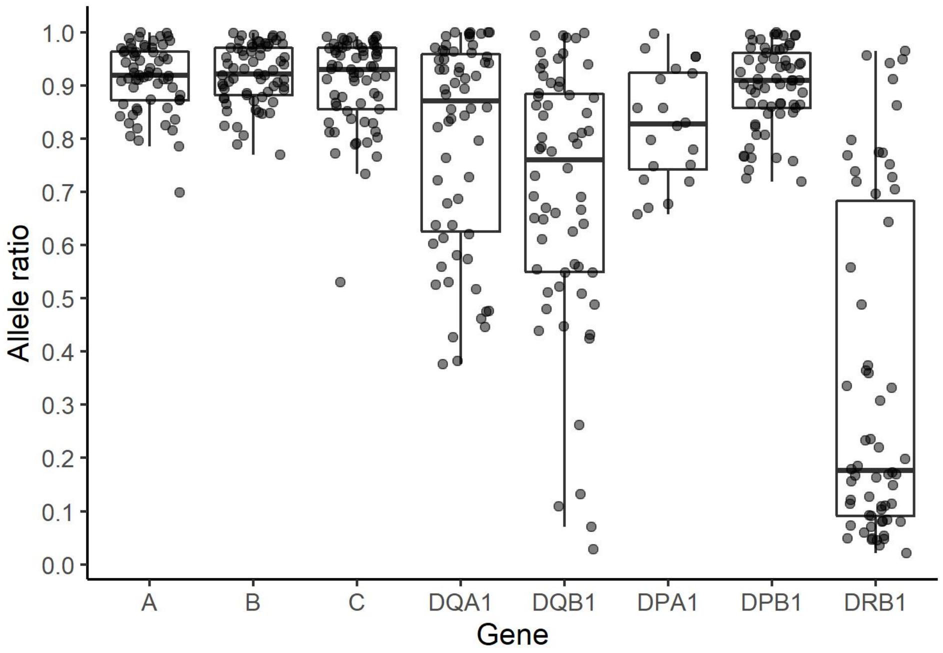

Supplementary Figure S3: Allele ratios of the eight genes of interest, excluding data for the two cases sequenced as part of preliminary experiments for method optimization. Only data from samples included in association analyses are shown. Allele ratios were calculated by dividing mean depth of coverage in exon 2 of the minor allele by the mean depth of coverage in exon 2 of the major allele. Homozygous typing results were excluded.

Allele	Cases	Tolerants	OR	[95% CI]	p-value
B*35:01	1 (2%)	10 (13%)	0.11	[0.0025 - 0.81]	0.023
C*04:01	1 (2%)	12 (16%)	0.089	[0.0020 - 0.63]	0.0063
C*07:04	4 (6%)	0 (0%)	∞	[0.83 - ∞]	0.039
DQA1*01:02	16 (26%)	8 (11%)	2.9	[1.1 - 8.6]	0.024
DQB1*05:01	4 (6%)	14 (18%)	0.31	[0.070 - 1.1]	0.044
DQB1*06:04	6 (10%)	1 (1%)	7.9	[0.92 - 370.]	0.045
DRB1*13:02	7 (11%)	1 (1%)	9.4	[1.2 - 430.]	0.022
DRB1*04:01	9 (15%)	2 (3%)	6.2	[1.2 - 61.]	0.013
DRB1 rare alleles	7 (11%)	25 (33%)	0.26	[0.088 - 0.69]	0.0041

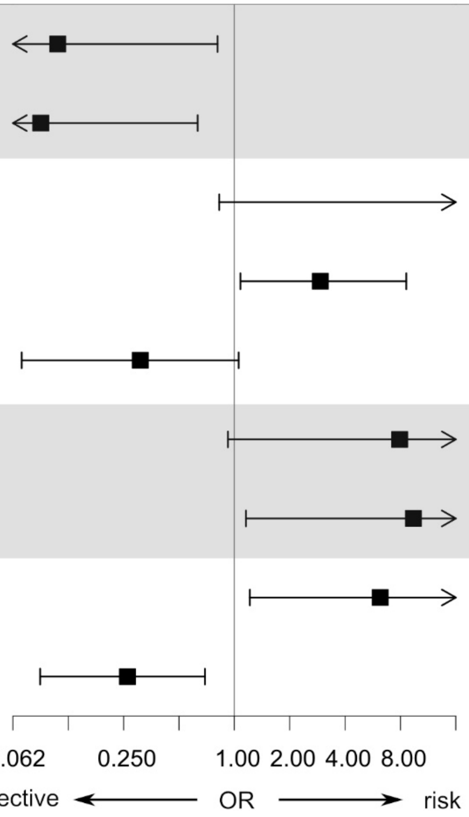

Supplementary Figure S4: Candidate alleles identified in the HTS-based discovery subset ($p < 0.05$) at two-field resolution. Alleles observed < 4 times were grouped into a “rare alleles” category for each gene. Odds ratios (OR), 95% confidence intervals, and unadjusted p-values are shown.

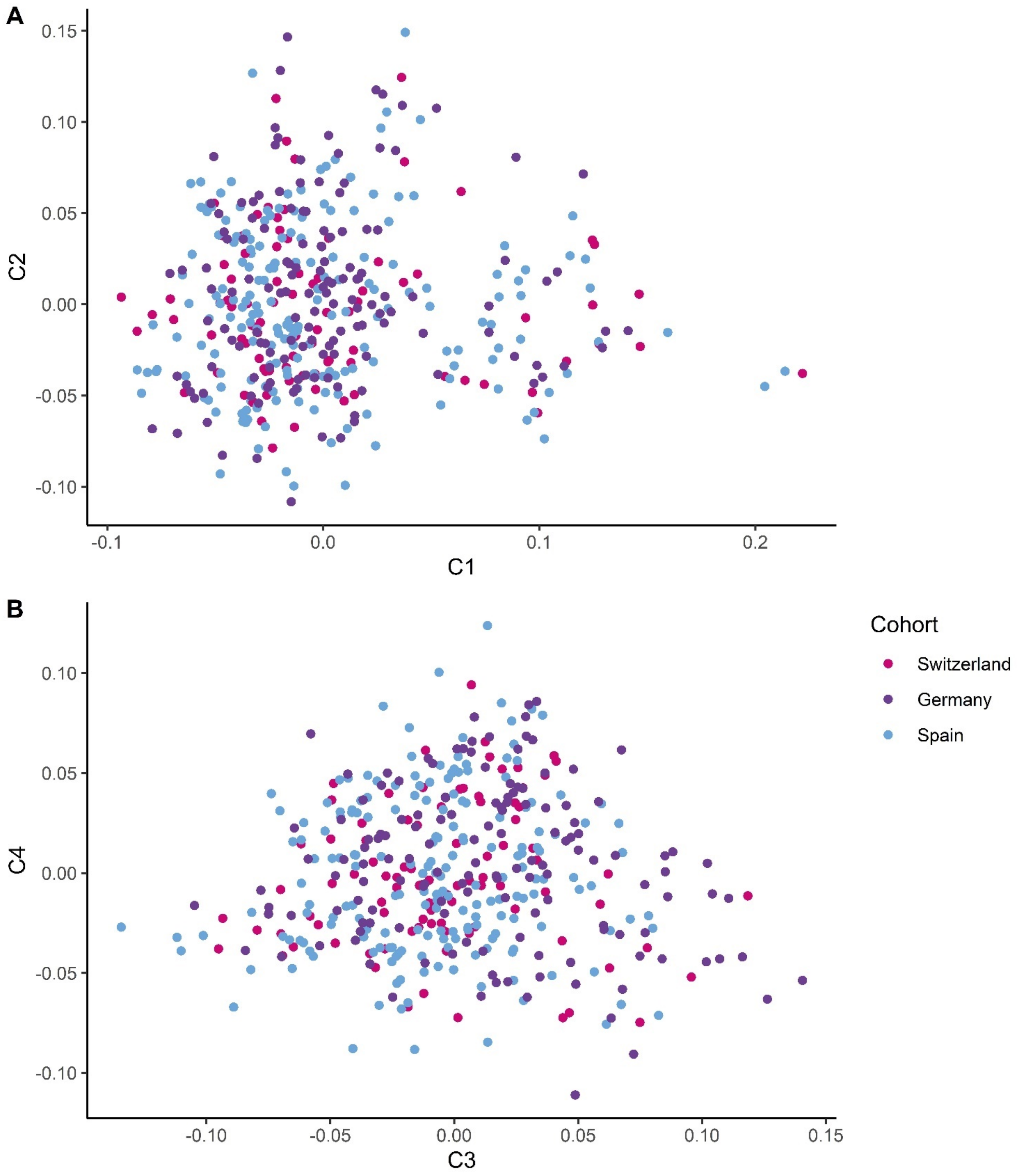

Supplementary Figure S5: (A) First two dimensions and (B) third and fourth dimension of the MDS analysis of HLA region SNPs in control samples of the three independent cohorts. Only data from samples included in association analyses are shown, i.e. excluding genetic outliers with non-European origin or related individuals, as described in the Methods.

2.2 Supplementary Tables

Gene	Primer	Sequence	Amplicon	Amplicon Amount used for pooling	Reference
HLA-A	F	ATCCTGGATACTCACGACGCGGAC	3398 bp	16.56 ng (S)	(Hosomichi et al., 2013)
	R	CATCAACCTCTCATGGCAAGAATTT			(Hosomichi et al., 2013)
HLA-B	F	AGGTGAATGGCTCTGAAAATTTGTCTC	4296 bp	20.94 ng (S)	(Hosomichi et al., 2013)
	R	AGAGTTTAATTGTAATGCTGTTTTGACACA			(Hosomichi et al., 2013)
HLA-C	F	GGCCGCCTGTACTTTTCTCAGCAG	4440 bp	21.65 ng (S)	(Hosomichi et al., 2013)
	R	CATGGTGAGTTTCCCTGTACAAGAG			(Hosomichi et al., 2013)
HLA-DQA1	F	GCAGACATGCACACACCAGAGA	5800 bp	28.28 ng (S)	
	R	TCATCATCATGCCACTTCCCAAT			
HLA-DQB1	F	TATGACAGCAATTTTCTCTCCCCTG	7494 bp	17.74 ng (L)	(Ehrenberg et al., 2017)
	R	TCATGTGCTTCTCTTGAGCAGTCTGA			(Hosomichi et al., 2013)
HLA-DPA1	F	TTGGCCTCTTGGCTATACCTCTTTT	9844 bp	23.31 ng (L)	(Shiina et al., 2012)
	R	GATCATCTGACTGTCTGGGATGG			
HLA-DPB1	F	TGGTCCAACAGGATCACATTTATAAGTGT	13410 bp	31.75 ng (L)	(Hosomichi et al., 2013)
	R	CCTGTAATGTGGGTGCAGATACCTC			
HLA-DRB1	F1.1	GCATCCACAGAATCACATTTCTAGTGTT	11899 -	154.2 ng (L)	(Hosomichi et al., 2013)
	F1.2.1	TCCACAGAATCACAGCATTTCTAGTGTT	15814 bp		(Ehrenberg et al., 2014, 2017)
	F1.3	GCATCCACAGAATCACATTTCCAGTATT			(Ehrenberg et al., 2014, 2017)
	F1.4.1	TCCACAGAATCACAGCATTTCCAGTATT			(Ehrenberg et al., 2014, 2017)
	R2.1	TGATTGACTTGCTGGCTGGTTTCTCATC			(Ehrenberg et al., 2014, 2017)

Supplementary Table S1: Primers used for long-range PCR, amplicon sizes and amount of purified PCR product added to the amplicon pool for the respective library preparation (shorter or longer genes, S and L libraries, respectively).

Gene	Cycles	PCR parameters*
HLA-A	30	98°C, 10 s; 68°C, 12 min
HLA-B	30	98°C, 10 s; 68°C, 12 min
HLA-C	30	98°C, 10 s; 68°C, 12 min
HLA-DPA1	30	98°C, 10 s; 68°C, 12 min
HLA-DPB1	30	98°C, 10 s; 68°C, 12 min
HLA-DRB1	30	98°C, 10 s; 58°C, 15 s; 68°C, 10 min
HLA-DQA1	25	98°C, 10 s; 68°C, 12 min
HLA-DQB1	30	98°C, 10 s; 52°C, 15 s; 68°C, 10 min

Supplementary Table S2: Long-range PCR cycling conditions for all genes of interest. *PCR cycling was preceded by a 2 min 94°C initial denaturation step and followed by a 10 min 68°C final extension step for all genes.

Issue checked	Action performed
Chromosome and position	Updated
Non-matching alleles	Removed
XY,Y,MT SNPs	Removed
Allele frequency (AF)	Removed if AF difference > 0.2
Strand	Updated
Palindromic SNPs	Removed if MAF > 0.4
Reference allele	Updated
Variant naming	Updated

Supplementary Table S3: Pre-imputation data checking. The pre-imputation checking program [3]compared markers and their frequencies to the HRC (Human Reference Consortium r1-1 2016) reference panel to check for potential issues using both location information and SNP names. Variants with no match (name or position) to the reference or failing to meet one of the above thresholds were listed for removal in PLINK. Mitochondrial (MT), X and Y-chromosomal (XY,Y) SNPs were excluded.

[3] https://www.well.ox.ac.uk/~wrayner/tools/#Checking

HLA-A allele	*HLA-Y* carrier	
	**no**	**yes**
*A*01:01:01*	16	2
*A*02:01:01*	32	3
*A*02:02:01*	1	0
*A*02:05:01*	1	2
*A*02:06:01*	1	0
*A*03:01:01*	18	2
*A*11:01:01*	8	0
*A*23:01:01*	1	0
*A*24:02:01*	9	2
*A*25:01:01*	3	0
*A*26:01:01*	3	1
*A*26:08*	0	1
*A*29:01:01*	0	1
*A*29:02:01*	1	0
*A*30:01:01**	0	5
*A*30:04:01*	1	0
*A*31:01:02**	1	4
*A*32:01:01*	9	0
*A*33:01:01*	0	1
*A*33:03:01**	0	1
*A*68:01:01*	2	0
*A*68:01:02*	5	1

Supplementary Table S4: Co-occurrence of *HLA-A* and *HLA-Y*, of which only the allele *HLA-Y*02:01* was typed, in the discovery subset using typing results obtained with HLA-HD. Only data from samples included in association analyses are shown. Alleles highlighted with an asterisk (*) have been reported to be associated with *HLA-Y*02:01* (Kawaguchi et al., 2017). Note: Most subjects are heterozygous for *HLA-A*, thus presence of *HLA-Y* also co-occurs with other alleles than those suggested to be associated.

Subject ID	Group	Gene	HLA-HD		NGSengine		PCR-SSP	
171_M	case	*DPB1*	*DPB1*124:01:01*	*DPB1*414:01:01*	***DPB1*02:01:02***	***DPB1*03:01:01***	NA	NA
198_T	tolerant	*DPB1*	***DPB1*04:01:01***	***DPB1*04:02:01***	*DPB1*126:01:01*	*DPB1*665:01*	NA	NA
251_T	tolerant	*DPB1*	*DPB1*124:01:01*	*DPB1*414:01:01*	***DPB1*02:01:02***	***DPB1*03:01:01***	NA	NA
477_T	tolerant	*DPB1*	*DPB1*124:01:01*	*DPB1*461:01*	***DPB1*02:01:02***	***DPB1*03:01:01***	NA	NA
404_M	case	*DQB1*	***DQB1*06:02:01***	***DQB1*03:02:01***	*DQB1*06:02:01*	-	NA	NA
476_T	tolerant	*DQB1*	***DQB1*06:02:01***	***DQB1*03:02:01***	*DQB1*06:02:01*	-	NA	NA
357_T	tolerant	*DRB1*	***DRB1*15:01:01***	*DRB1*11:04:01*	NA	NA	*DRB1*15*	***DRB1*07***
480_T	tolerant	*DRB1*	***DRB1*16:01:01***	-	NA	NA	*DRB1*16*	***DRB1*07***

Supplementary Table S5: HTS-based HLA typing results that were identified as suspicious in the quality control. Typing results that were ultimately accepted and used for association analyses are highlighted in bold. Note: for the two samples with allelic dropout of *HLA-DRB1*07*, the typing result was accepted as *–DRB1*07:01:01* in order not to introduce a new allele category for subsequent association analyses as this was the only *–DRB1*07* allele typed in the discovery subset.

3 References

Ehrenberg, P. K., Geretz, A., Baldwin, K. M., Apps, R., Polonis, V. R., Robb, M. L., et al. (2014). High-throughput multiplex HLA genotyping by next-generation sequencing using multi-locus individual tagging. *BMC Genomics* 15, 864. doi:10.1186/1471-2164-15-864.

Ehrenberg, P. K., Geretz, A., Sindhu, R. K., Vayntrub, T., Fernández Viña, M. A., Apps, R., et al. (2017). High-throughput next-generation sequencing to genotype six classical HLA loci from 96 donors in a single MiSeq run. *HLA*. doi:10.1111/tan.13133.

Gogarten, S. M., Bhangale, T., Conomos, M. P., Laurie, C. A., McHugh, C. P., Painter, I., et al. (2012). GWASTools: an R/Bioconductor package for quality control and analysis of genome-wide association studies. *Bioinformatics* 28, 3329–3331. doi:10.1093/bioinformatics/bts610.

González-Galarza, F. F., Takeshita, L. Y. C., Santos, E. J. M., Kempson, F., Maia, M. H. T., Da Silva, A. L. S., et al. (2015). Allele frequency net 2015 update: New features for HLA epitopes, KIR and disease and HLA adverse drug reaction associations. *Nucleic Acids Res.* doi:10.1093/nar/gku1166.

Hosomichi, K., Jinam, T. A., Mitsunaga, S., Nakaoka, H., and Inoue, I. (2013). Phase-defined complete sequencing of the HLA genes by next-generation sequencing. *BMC Genomics* 14, 1. doi:10.1186/1471-2164-14-355.

Kawaguchi, S., Higasa, K., Shimizu, M., Yamada, R., and Matsuda, F. (2017). HLA-HD: An accurate HLA typing algorithm for next-generation sequencing data. *Hum. Mutat.* doi:10.1002/humu.23230.

Kotsch, K., and Blasczyk, R. (2000). The Noncoding Regions of HLA-DRB Uncover Interlineage Recombinations as a Mechanism of HLA Diversification. *J. Immunol.* 165, 5664–5670. doi:10.4049/jimmunol.165.10.5664.

Langmead, B., and Salzberg, S. L. (2012). Fast gapped-read alignment with Bowtie 2. *Nat. Methods* 9, 357–359. doi:10.1038/nmeth.1923.

Maiers, M., Gragert, L., and Klitz, W. (2007). High-resolution HLA alleles and haplotypes in the United States population. *Hum. Immunol.* doi:10.1016/j.humimm.2007.04.005.

Robinson, J., Halliwell, J. A., Hayhurst, J. D., Flicek, P., Parham, P., and Marsh, S. G. E. (2015). The IPD and IMGT/HLA database: Allele variant databases. *Nucleic Acids Res.* doi:10.1093/nar/gku1161.

Shiina, T., Suzuki, S., Ozaki, Y., Taira, H., Kikkawa, E., Shigenari, A., et al. (2012). Super high resolution for single molecule-sequence-based typing of classical HLA loci at the 8-digit level using next generation sequencers. *Tissue Antigens*. doi:10.1111/j.1399-0039.2012.01941.x.
